# Urachal Carcinoma with Peritoneal Dissemination Treated with Chemotherapy and Surgical Resection Leading to Prolonged Survival with No Recurrence

**DOI:** 10.1155/2018/9836154

**Published:** 2018-06-13

**Authors:** Masato Yasui, Ryosuke Jikuya, Tomoyuki Tatenuma, Kentaro Muraoka, Susumu Umemoto, Masaki Kawai, Tsutomu Kouno, Takeshi Kishida

**Affiliations:** ^1^Department of Urology, Kanagawa Cancer Center, Yokohama, Japan; ^2^Department of Medical Oncology, Sasaki Foundation Kyoundo Hospital, Tokyo, Japan

## Abstract

A 56-year-old man was admitted to our hospital for urachal carcinoma with peritoneal dissemination. He received first-line chemotherapy with gemcitabine and cisplatin. After the fifth cycle, a computed tomography (CT) scan revealed abdominal fluid, and his serum tumor marker levels were increased. The patient was started on second-line therapy with FOLFIRI. After 11 cycles, his tumor decreased in size and no new metastatic lesions were detected. The patient underwent complete tumor resection with partial cystectomy and pelvic lymph node dissection. The tumor was removed, along with adhering surrounding organs, including the omentum, peritoneum, abdominal rectus muscle, and vermiform appendix. Although pathological examination confirmed peritoneal dissemination, his tumor markers normalized soon after surgery. The patient has survived 62 months after surgery without any adjuvant therapy and with no evidence of recurrence. To our knowledge, this is the longest duration of survival without recurrence of a patient with urachal carcinoma with peritoneal dissemination who received multimodal therapy.

## 1. Introduction

Urachal carcinoma is a rare malignant tumor, accounting for fewer than 1% of all primary bladder cancers [1]. Because tumor growth is asymptomatic, urachal carcinoma usually presents at an advanced stage. Surgery has been shown to be effective for local disease, but there is no confirmed treatment for metastasis.

Peritoneal dissemination is a sign of aggressive disease and an indicator of poor survival [2]. The rarity of these tumors makes it difficult to gather evidence-based treatment from prospective trials. To our knowledge, only single institution studies and case reports have been published.

This report describes a patient with urachal carcinoma with peritoneal dissemination who was treated with chemotherapy and surgical resection resulting in prolonged survival without recurrence.

## 2. Case Report

**Figure 1:**
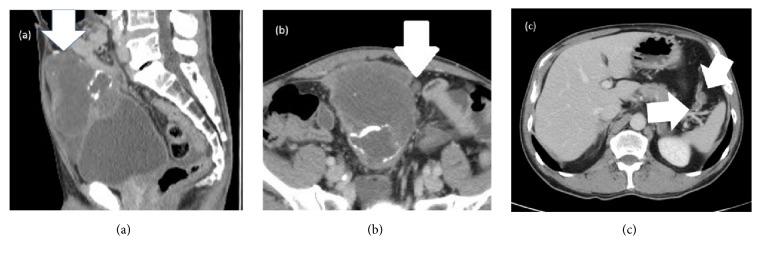
Abdominal computed tomographic image taken before systemic chemotherapy, showing (a) a 9-cm mass extending from the bladder to the umbilicus (white arrow) and ((b) and (c)) intraperitoneal nodules (white arrows).

**Figure 2:**
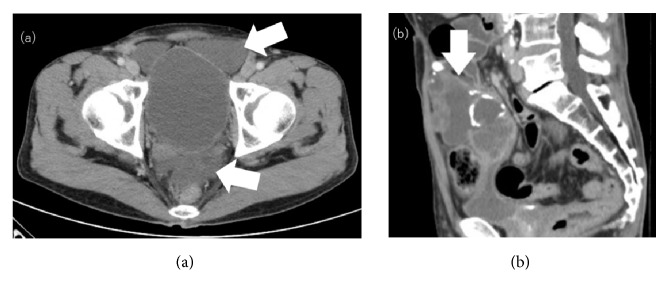
(a) Abdominal computed tomographic image taken after five cycles of gemcitabine and cisplatin (GC). Abdominal fluid was detected (white arrows). (b) Abdominal computed tomographic image taken after FOLFIRI (*ℓ*-leucovorin + 5-fluorouracil + irinotecan). The tumor size had decreased to 7 cm (white arrow).

**Figure 3:**
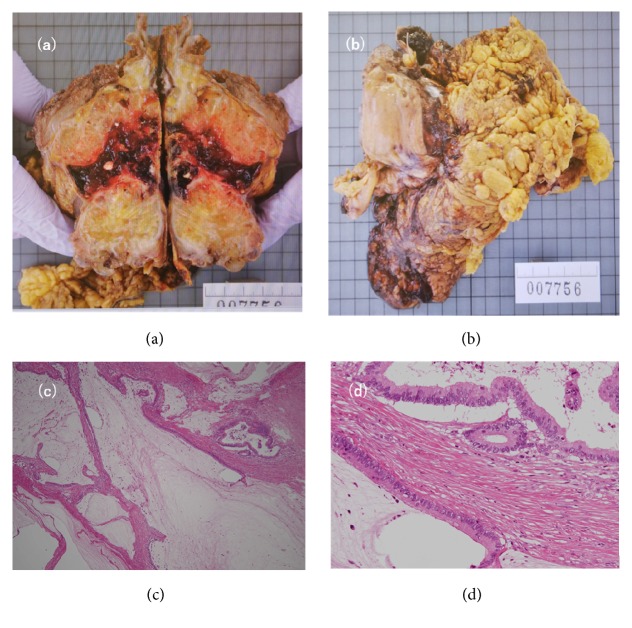
(a) and (b) show surgical specimen of the urachal carcinoma. The tumor was removed, along with surrounding adherent organs, including the omentum, peritoneum, and bladder dome. Disseminated nodules were detected in the omentum. (c) and (d) show cords of adenocarcinoma cells in abundant mucin (H&E stain).

**Figure 4:**
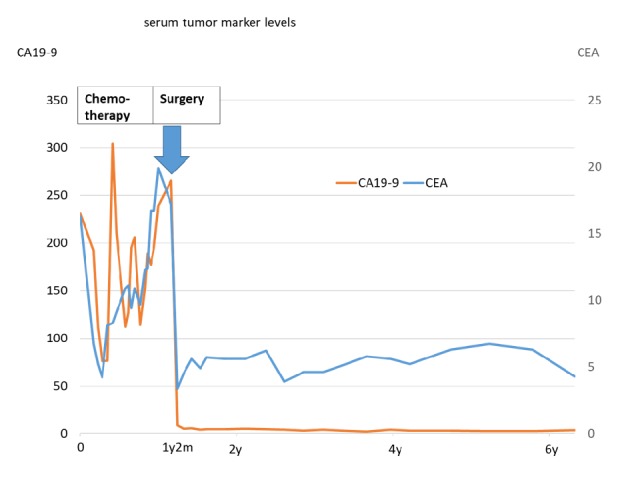
Serum tumor marker concentrations. Serum concentrations of carbohydrate antigen 19-9 (CA19-9; orange line) and carcinoembryonic antigen (CEA; blue line) were high during chemotherapy but normalized dramatically after surgery, with no significant increase detected to date.

A 56-year-old man was admitted to our hospital after resection of a lymph node in his groin revealed adenocarcinoma. Contrast-enhanced computed tomography (CT) showed a 9-cm mass extending from the bladder to the umbilicus, along with intraperitoneal nodules suggesting peritoneal dissemination (Figures 1(a), 1(b), and 1(c)). Cystoscopy showed an extrinsic mass located on the dome. Serum assays showed high levels of carcinoembryonic antigen (CEA), to 16.3 ng/mL, and carbohydrate antigen 19-9 (CA19-9), to 230.9 U/mL. The patient was diagnosed with urachal carcinoma with suspected peritoneal dissemination and was started on systemic chemotherapy with intravenous gemcitabine (1000 mg/m^2^ on days 1 and 8 of each 21-day cycle) plus cisplatin (70 mg/m^2^ on day 2 of each cycle). After two cycles, a CT scan showed no marked changes in the lesion; after four cycles, his serum CEA and CA19-9 concentrations had decreased to 4.2 ng/mL and 76.1 U/mL, respectively. However, after five cycles, his CEA concentration had increased to 8.3 ng/mL and his CA19-9 concentration had also increased to 304.1 ng/mL with a CT scan showing changes in the tumor and the appearance of abdominal fluid (Figure 2(a)). Because of the considered histological and clinical similarities between colorectal and urachal carcinoma, his treatment was changed to FOLFIRI (i.v. infusion of 180 mg/m^2^ irinotecan, 200 mg/m^2^  *ℓ*-leucovorin, and 400 mg/m^2^ 5-fluorouracil (5-FU) on day 1 of each 14-day cycle, followed by continuous infusion of 2400 mg/m^2^ 5-FU for 46 hours) after receiving informed consent. After 11 cycles of FOLFIRI, serum tumor marker levels had not changed markedly, but a CT scan showed a reduction in tumor size to 7 cm (Figure 2(b)) and no new distant metastases. Because chemotherapy was able to suppress tumor progression, a surgical approach was chosen, and complete resection of the tumor, along with partial cystectomy and pelvic lymph node dissection, was performed. The tumor was found to adhere to surrounding organs with mucinous fluid, with disseminated nodules present in the greater omentum. The tumor was removed, along with surrounding adherent organs, including the bladder dome, omentum, peritoneum, abdominal rectus muscle, and vermiform appendix. Cytology showed that the mucinous fluid in the abdominal cavity was class V and the final pathological diagnosis was urachal mucinous adenocarcinoma with invasion of the omentum and peritoneal dissemination (Figure 3). There were no lymph node metastases, and surgical margins were negative. The patient was discharged from the hospital 14 days after surgery with no complications. Soon after surgery, his serum tumor markers had normalized. Tumor marker levels were measured every month and CT scans performed periodically until six months after surgery. Subsequent follow-up included CT scans and tumor marker level measurement every three months for the first three years and every six months thereafter. At the time of writing this report, i.e., 62 months after surgery and without any adjuvant chemotherapy, the patient remains alive. Although he showed a slight increase in serum CEA level to 6.3 ng/mL, his serum CA19-9 level remained within normal limits (Figure 4), and no radiological recurrence has been detected.

## 3. Discussion

Urachus is a fibrous remnant of the urogenital sinus and allantois. Generally, the urachus involutes after the third trimester and forms the medium umbilical ligament. In about one-third of adults, however, urachal remnants persist as a tubular or cystic structure and may give rise to urachal carcinoma [7].

The survival rate of patients with urachal carcinoma is relatively poor, with 5-year overall survival rates reported to be less than 50% [8]. Poor survival outcomes are associated with the high frequency of diagnosis during the late stages of the disease, as typical symptoms such as gross hematuria do not occur until the tumor invades the bladder [9]. Moreover, tumors not invading the bladder are frequently detected during the advanced stage based on the symptoms associated with widespread metastasis. The median survival of patients with metastatic disease has been reported to be less than 24 months [10]. In contrast, patients with early-stage urachal carcinoma have a much better prognosis [2, 11]. Treatment strategies are therefore needed for patients with advanced stage disease.

Although systemic chemotherapy may be effective in treating patients with metastatic disease, no standard chemotherapy regimens have been developed yet, and various chemotherapeutic agents have been used [12]. Cisplatin-based regimens designed for bladder cancer have been used in many patients with urachal adenocarcinoma [13], but their effectiveness is limited. The histological and clinical similarity of urachal to colonic adenocarcinoma suggests that regimens that include 5-FU may be effective [14]. A comparison of cisplatin-based, 5-FU-based, and 5-FU + cisplatin-based regimens found that the combination had the highest response rate and the lowest progression rate [8]. Our patient was initially treated with GC (gemcitabine and cisplatin) and subsequently with FOLFIRI, a 5-FU based regimen, resulting in partial tumor shrinkage that enabled curative surgery. GC was chosen as the first-line treatment because, at the time of initiation of the treatment, many reports of various regimens including GC had been published, but none of these studies compared the effectiveness of 5-FU-based regimens and GC. The effectiveness of 5-FU-based regimens has recently been shown, and these regimens should be considered as the first-line therapy. Surgery is the standard treatment for localized disease. As positive tumor margins are significantly associated with poor prognosis [15], wide excision with partial or radical cystectomy and en bloc resection of the umbilical ligament up to the umbilicus have been recommended [9]. The need to surgically resect metastatic sites is uncertain. However, if these metastases are resectable and not growing rapidly, surgery may enhance survival [9, 16].

In general, malignant tumors with peritoneal dissemination are associated with poor prognosis. However, a small number of patients with urachal adenocarcinoma, including our case, have survived for a certain period after surgery.  Table 1 contains a list of reports of urachal adenocarcinoma with peritoneal dissemination with cytoreductive surgery. Although Sugarbaker reported a patient who survived 11 years after diagnosis [4], our case had the longest reported survival without recurrence. Sugarbaker's patient had undergone three rounds of cytoreductive surgery and hyperthermic intraperitoneal chemotherapy when recurrence was detected. In another case, no recurrence was detected ten months after surgery with adjuvant systematic chemotherapy [6]. The therapeutic effect of multimodal treatment in urachal adenocarcinoma with peritoneal dissemination remains uncertain. Based on the results of our report and other reports with long survival times, mucinous adenocarcinoma may have a strong tendency for local invasion which results in peritoneal dissemination; however, the frequency of distant metastasis is low. Consequently, if peritoneal dissemination is detected with no distant metastasis, removal of the tumor with wide margins may be a curative option in mucinous urachal adenocarcinoma. Moreover, neoadjuvant chemotherapy may be effective in aborting tumor growth, and the likelihood of complete resection could increase.

## 4. Conclusion

Despite presenting with peritoneal dissemination, urachal adenocarcinoma in our patient was controlled with systemic chemotherapy allowing complete resection of the visible tumor. Pathological examination of the surgical specimen revealed disseminated cancer in the peritoneum, a factor usually associated with poor prognosis. Neoadjuvant treatment plus surgery resulted in prolonged survival with no recurrence, despite the absence of adjuvant treatment. Recurrence of urachal carcinomas is mostly detected within 2 years after surgery [10]; however, these tumors may occur after 2 years, requiring careful monitoring. Our findings suggest that multimodal therapy may be curative for advanced urachal carcinoma.

## Figures and Tables

**Table 1:** Previous reports of urachal adenocarcinoma with peritoneal dissemination treated with cytoreductive surgery.

Author	Age	Gender	pre-operative therapy	HIPEC	post-operative therapy	time to recurrence (months)	Post-recurrence therapy	time to death (months)
Krane LS et al. [3]	unknown	M	unknown	Mitomycin C	unknown	11	5-FU	21
	unknown	M	unknown	Mitomycin C	unknown	31	carbolpatin, FOLFORI	87

Sugarbaker PH et al. [4]	32	F	none	EPIC	none	24	surgery + HIPEC	132
			none	Mitomycin C	none	NED at 20 months	-	-

Martinez A et al. [5]	32	M	none	oxaliplatin	none	NED at 24 months	-	-

Ozawa M et al. [6]	48	M	none	none	TS-1 + cisplatin	NED at 10 months	-	-

Yasui M et al.^*∗*^	56	M	GC / FOLFIRI	none	none	NED at 62months	-	-

HIPEC, hyperthermic intraperitoneal chemotherapy; EPIC, early postoperative intraperitoneal chemotherapy; GC, gemcitabine + cisplatin, FOLFIRI, *ℓ*-leucovorin + 5-flourouracil + irinotecan; NED, no evidence of disease

^*∗*^Present study

## References

[1] Bruins H. M., Visser O., Ploeg M. (2012). The clinical epidemiology of urachal carcinoma; results of a large, population-based study. *The Journal of Urology*.

[2] Molina J. R., Quevedo J. F., Furth A. F., Richardson R. L., Zincke H., Burch P. A. (2007). Predictors of survival from urachal cancer: a Mayo Clinic study of 49 cases. *Cancer*.

[3] Krane L. S., Kader A. K., Levine E. A. (2012). Cytoreductive surgery with hyperthermic intraperitoneal chemotherapy for patients with peritoneal carcinomatosis secondary to urachal adenocarcinoma. *Journal of Surgical Oncology*.

[4] Sugarbaker P. H., Verghese M., Yan T. D., Brun E. (2008). Management of mucinous urachal neoplasm presenting as pseudomyxoma peritonei. *TUMORI*.

[5] Martínez A., Ferron G., Mery E., Gladieff L., Delord J. P., Querleu D. (2012). Peritoneal pseudomyxoma arising from the urachus. *Surgical Oncology*.

[6] Ozawa M., Kuromoto A., Morozumi K. (2017). Two cases of urachal carcinoma treated by TS-1/CDDP as adjuvant chemotherapy. *Hinyokika Kiyo*.

[7] Schubert G. E., Pavkovic M. B., Bethke-Bedurftig B. A. (1982). Tubular urachal remnants in adult bladders. *The Journal of Urology*.

[8] Szarvas T., Módos O., Niedworok C. (2016). Clinical, prognostic, and therapeutic aspects of urachal carcinoma—a comprehensive review with meta-analysis of 1,010 cases. *Urologic Oncology: Seminars and Original Investigations*.

[9] Siefker-Radtke A. (2012). Urachal Adenocarcinoma: a clinician's guide for treatment. *Seminars in Oncology*.

[10] Hayashi T., Yuasa T., Uehara S. (2016). Clinical outcome of urachal cancer in Japanese patients. *International Journal of Clinical Oncology*.

[11] Siefker-Radtke A. O., Gee J., Shen Y. (2003). Multimodality management of urachal carcinoma: the M. D. Anderson Cancer Center experience. *The Journal of Urology*.

[12] Yazawa S., Kikuchi E., Takeda T. (2012). Surgical and chemotherapeutic options for urachal carcinoma: Report of ten cases and literature review. *Urologia Internationalis*.

[13] Miyata Y., Sagara Y., Matsuo T. (2011). Response of recurrent urachal cancer to gemcitabine and cisplatin therapy: A case report and literature review. *Anticancer Reseach*.

[14] Kanamaru T., Iguchi T., Yukimatsu N. (2015). A case of metastatic urachal carcinoma treated with FOLFIRI (irinotecan and 5-fluorouracil/leucovorin) plus bevacizumab. *Urology Case Reports*.

[15] Behrendt M. A., De Jong J., Van Rhijn B. W. (2016). Urachal cancer: contemporary review of the pathological, surgical, and prognostic aspects of this rare disease. Minerva Urol Nefrol. *Minerva Urologica e Nefrologica*.

[16] Kawakami S., Kageyama Y., Yonese J. (2001). Successful treatment of metastatic adenocarcinoma of the urachus: report of 2 cases with more than 10-year survival. *Urology*.

